# Serum ionised calcium, thrombin time (TT), fibrinogen (FIB) after heparin and sodium citrate anticoagulation in children with sepsis

**DOI:** 10.5937/jomb0-56286

**Published:** 2025-09-05

**Authors:** Huixia Wang, Hongfang Zhao

**Affiliations:** 1 Northwest Women's and Children's Hospital, Department of Pediatric Emergency and Intensive Care Unit, Xi'an, Shaanxi Province, China; 2 Air Force Medical University, Tangdu Hospital, Department of Paediatrics, Xi'an, Shaanxi Province, China

**Keywords:** serum ionised calcium (iCa2+), thrombin time (TT), fibrinogen (FIB), heparin anticoagulation, sodium citrate anticoagulation, children with sepsis, continuous blood purification, anticoagulation risk, bleeding risk, serumski jonizovani kalcijum (iCa2+), trombinsko vreme (TT), fibrinogen (FIB), antikoagulacija heparinom, antikoagulacija natrijum-citratom, deca sa sepsom, kontinuirano prečiščavanje krvi, rizik od antikoagulacije, rizik od krvarenja

## Abstract

**Background:**

This study aimed to compare the anticoagulant efficacy and bleeding risk of systemic heparin versus local sodium citrate anticoagulation in continuous renal replacement therapy (CRRT) for children with sepsis in the intensive care unit (ICU).

**Methods:**

Clinical data from 96 children with sepsis admitted to the ICU of Northwest Women's and Children's Hospital between January 2021 and January 2024 were retrospectively analysed. The children were divided into a control group (CG) and an observation group (OG). Coagulation parameters, the effectiveness of blood purification treatment, and the incidence of bleeding events during CRRT were compared between the two groups.

**Results:**

After treatment, significant increases were observed in activated partial thromboplastin time (APTT), prothrombin time (PT), and thrombin time (TT), while fibrinogen (FIB) levels decreased. APTT, PT, and TT showed more significant increases in the OG, and FIB levels were notably reduced. pH, HCO3-, and Na+ levels were significantly different after treatment, and creatinine (Cr) and urea nitrogen (BUN) levels were lower in both groups, with the OG showing a slightly more significant decrease in Cr and BUN compared to the CG. Ionised calcium (iCa2+) levels were higher in the OG than in the CG. The bleeding incidence in the OG was 27.08%, lower than the 35.42% observed in the CG (all P<0.05).

**Conclusions:**

Local sodium citrate anticoagulation demonstrated a favourable anticoagulation effect with a lower bleeding risk than systemic heparin anticoagulation in CRRT for children with sepsis in the ICU.

## Abstract (sr)

**Uvod:**

Cilj ove studije bio je da uporedi efikasnost antikoagulacije i rizik od krvarenja kod sistemske antikoagulacije heparinom u odnosu na lokalnu antikoagulaciju natrijum-citratom tokom kontinuirane zamene bubrežne funkcije (CRRT) kod dece sa sepsom na odeljenju intenzivne nege (ICU).

**Metode:**

Retrospektivno su analizirani klinički podaci 96 dece sa sepsom primljenih na ICU Bolnice za žene i decu severozapadne Kine u periodu od januara 2021. do januara 2024. Deca su podeljena u kontrolnu grupu (CG) i posmatranu grupu (OG). Upoređeni su parametri koagulacije, efikasnost tretmana prečišćavanja krvi i učestalost krvarenja tokom CRRT-a između dve grupe.

**Rezultati:**

Nakon tretmana, uočena su značajna povećanja aktiviranog parcijalnog tromboplastinskog vremena (APTT), protrombinskog vremena (PV) i trombinskog vremena (TT), dok su nivoi fibrinogena (FIB) opali. U OG su APTT, PT i TT pokazali izraženije povećanje, dok su nivoi FIB bili značajnije smanjeni. Nivoi pH, HCO3i Na+ su se značajno razlikovali nakon tretmana, a vrednosti kreatinina (Cr) i azotne ureje (BUN) su bile niže u obe grupe, pri čemu je u OG zabeležen nešto veći pad Cr i BUN u poređenju sa CG. Nivoi jonizovanog kalcijuma (iCa2+) bili su viši u OG nego u CG. Incidenca krvarenja u OG iznosila je 27,08%, što je bilo niže u poređenju sa 35,42% u CG (svi P<0,05).

**Zaključak:**

Lokalna antikoagulacija natrijum-citratom pokazala je povoljan antikoagulantni efekat sa nižim rizikom od krvarenja u odnosu na sistemsku antikoagulaciju heparinom tokom CRRT-a kod dece sa sepsom na odeljenju intenzivne nege.

## Introduction

Sepsis is one of the common conditions in the pediatric ICU, and it is also a serious systemic infection reaction. It is usually caused by bacterial infection but may also be caused by other microbial infections [1] [2]. When the body responds to infection, the immune system will release many inflammatory mediators, leading to systemic inflammatory response. This inflammatory response may lead to vasodilation, blood coagulation, microcirculation disturbance, and organ function damage and may lead to multiple organ failure (MOF) and death in severe cases [3] [4]. Early recognition and rapid intervention are key to treating sepsis, including antibiotic treatment, fluid resuscitation, blood purification, and supportive treatment [5].

CRRT, as one of the essential means of sepsis treatment, can effectively remove inflammatory mediators and metabolites in the blood, improve the condition, and improve the survival rate of children [6] [7]. However, during CRRT, anticoagulation is the key to ensuring the smooth progress of treatment. Still, anticoagulation also brings the risk of bleeding, especially for children with sepsis who are at high risk of bleeding [8]. Systemic heparin and local sodium citrate are commonly used anticoagulant methods. Still, there is a lack of systematic research and comparison on the anticoagulant effect and bleeding risk of CRRT in children with sepsis. Systemic heparin anticoagulation has been considered a commonly used anticoagulation method in continuous blood purification [9] [10]. Heparin prevents the coagulation reaction by inhibiting the thrombin activity, thus achieving the anticoagulant effect. However, systemic application of heparin anticoagulation may increase the risk of bleeding, especially in inflammatory conditions such as sepsis. Therefore, for the anticoagulation effect of continuous blood purification in children with sepsis in the ICU, further clinical studies are needed to evaluate the efficacy of systemic heparin anticoagulation. In recent years, applying local sodium citrate anticoagulation in continuous blood purification has received extensive attention [11]. Sodium citrate inhibits the activity of thrombin by binding with calcium ions, achieving the effect of local anticoagulation and thereby reducing the risk of bleeding caused by systemic anticoagulation [12]. Studies have shown that local sodium citrate anticoagulation has a good anticoagulation effect in continuous blood purification and may have a lower risk of bleeding compared with systemic heparin anticoagulation [13]. However, there is a lack of sufficient research data to support the anticoagulation effect of children with sepsis in the ICU, and further research is needed to evaluate its applicability and safety in this specific population.

This study compared the anticoagulant efficacy and bleeding risk associated with systemic heparin and local sodium citrate anticoagulation during continuous renal replacement therapy (CRRT) in children with sepsis in the intensive care unit (ICU). The goal was to identify a more effective and safer anticoagulation strategy for CRRT in this vulnerable patient population.

## Materials and methods

### Subjects

The clinical data of 96 children with sepsis in ICU treated in Northwest Women’s and Children’s Hospital from January 2021 to January 2024 were retrospectively analysed. Different anticoagulation regimens divided the children into CG (systemic heparin) and OG (local sodium citrate), with 48 cases each. In the OG, there were 21 males and 27 females, 1–10 years old (7.86±3.51), body weight of 7–31 kg. In the CG, there were 23 males and 25 females aged 1 to 11 (6.84±2.17), with a body weight of 6–28 kg. The clinical data of the two groups were comparable (>0.05). The families of the children voluntarily signed the informed consent form. It was approved by the Northwest Women’s and Children’s Hospital ethics committee.

Inclusion criteria: (1) children meeting the diagnostic criteria of *Guidelines for Inflammatory Sepsis/Septic Shock* (2014) [14]; (2) patients requiring continuous blood purification therapy. (3) sepsis was clinically diagnosed and treated in ICU; (4) the monitoring data of vital signs of children are stable, and they have the tolerance of surgery and treatment; (5) the patient and the legal guardian agreed to participate in the trial and signed the informed consent.

Exclusion criteria: (1) patients with severe mental illness or intellectual disability, affecting the understanding and cooperation of the intervention measures; (2) There is an obvious risk of thrombosis, such as recent deep vein thrombosis (DVT) or pulmonary embolism; (3) patients who have received emotional nursing combined with targeted interventions used in similar studies; (4) a history of heparin or sodium citrate allergy; (5) severe liver and kidney dysfunction; (6) other essential comorbidities or clinical conditions, such as severe anaemia, malignant tumour, serious cardiovascular disease, which may affect the interpretation of the study results.

### Treatment

The blood purifier was an Aquarius 3.5 CRRT machine (America Baxter), and the treatment mode was continuous veno-venous hemofiltration (CVVH). Before the cardiopulmonary bypass, the internal jugular vein or femoral vein indwelling needle double-lumen catheter was usually used for vascular access. A filter was required for blood purification during cardiopulmonary bypass. New filters were used for each treatment. The replacement fluid speed was 2,000–4,000 mL/h, and the treatment time was 24 h/d. The blood flow was generally set at 200 mL/min, but for some patients, it may not reach the set blood flow, which can be adjusted according to the actual situation. The duration of cardiopulmonary bypass treatment was generally 10–12 h, which was adjusted according to the specific situation of patients and treatment needs. The basic replacement fluid for hemofiltration was purchased from Qingshan Likang Pharmaceutical Co., Ltd. (4 L/bag, with glucose 10.6 mmol/L, chloride ion 118 mmol/L, magnesium 0.797 mmol/L, calcium 1.60 mmol/L, sodium 113 mmol/L. In addition, it contained auxiliary hydrochloric acid or NaCl as pH adjustment). The filter is multiflow 100 (filter area is 1.6 m^2^).

In the OG, 4% sodium citrate solution (Sichuan Nigale Biomedical Co., Ltd., batch No.: 13145) was pumped simultaneously with an infusion pump at a speed of 180–220 mL/h and a blood flow of 150–180 mL/min; 10% calcium gluconate (Sichuan MeidaKang Huakang Pharmaceutical Co., Ltd., batch No.: 1804021) was infused at the venous end of the blood vessel at a blood flow rate of 20 mL/h. The rate of calcium supplementation was adjusted according to the level of ionised calcium in the body, and the level of serum ionised calcium in the body was not less than 0.9 mmol/L.

The CG: no heparin anticoagulation, blood flow 200–250 mL/min while flushing 100 mL/h. Before flushing, the arterial pipeline was blocked, and the ultrafiltration and flushing volumes should be calculated.

In the treatment process, according to the patient’s specific situation, the dewatered amount needed to be adjusted to maintain the patient’s fluid balance and internal environment stability.

### Anticoagulation effect detection

### Serum ionised calcium

The blood sample was drawn: the pipeline extraction before the filter – before the citric acid input, the iCa^2+^ level in the body was reflected; the pipeline extraction after the filter – before calcium supplementation and after the filter, the iCa^2+^ level in vitro was reflected.

### aPTT

Two mL of whole venous blood samples were collected and injected into an ACT tube with an inner diameter of 8 mm containing 12 mg diatomite. After thoroughly mixing the blood and diatomite, the ACT detector was used for timing. The detector stopped and gave an alarm when the blood was clotted, and the ACT value (seconds) appeared. The sampling before the filter reflected the coagulation state in the body, and the sampling position was in the pipeline before the citric acid input. The sampling after the filter reflected the in vitro coagulation state, and the sampling position was selected in the pipeline after the filter and before calcium supplementation.

### Observation of blood coagulation

The coagulation changes of the filter and venous chamber were observed. The coagulation degree of filter fibres was divided into four levels [15]: Grade 0 refers to no coagulation or only a small amount of fibre coagulation; Grade 1 refers to more than half of coagulation or bleeding into bundles of fibre coagulation; Grade II indicates that more than half of the fibres have been coagulated; Grade III refers to serious coagulation requiring replacement of the dialyser or clear increase in transmembrane pressure of the dialyser. According to the coagulation of venous chamber coagulation, it was divided into three levels: no coagulation, a little coagulation, and obvious coagulation. Grade 0–1 indicates a good anticoagulation effect, and Grade 2–3 indicates a poor one.

### Observation index

(1) The blood pH, HCO_3_^-^, Na^+^, and other biochemical indexes were analyzed.

(2) The coagulation function, including FIB, TT, PT, and APTT, was compared.

(3) Aggravated bleeding was observed.

(4) The changes in Cr and BUN were observed.

### Statistical analysis

Data were analysed using SPSS version 19.0. Continuous variables are presented as mean ± SD and compared using independent t-tests for normally distributed data or Mann-Whitney U tests for non-normally distributed data. Categorical variables are presented as percentages and analysed using the Chi-square or Fisher’s exact test. Paired comparisons of pre- and post-treatment values were made using paired t-tests or Wilcoxon signed-rank tests. A p-value of <0.05 was considered statistically significant.

## Results

### Primary diseases

**Figure 1:**
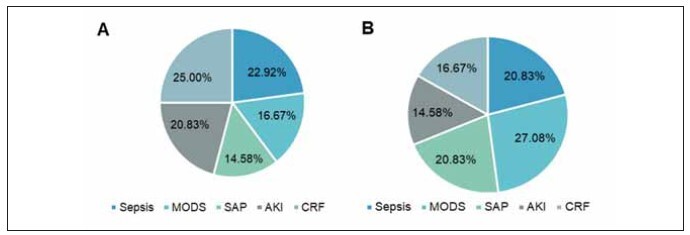
Primary diseases.

In the OG, sepsis was observed in 11 subjects (22.92%), multiple organ failure in 8 subjects (16.67%), severe pancreatitis in 7 subjects (14.58%), acute kidney injury (AKI) in 10 subjects (20.83%), and chronic renal insufficiency in 12 subjects (25.0%). In the CG, sepsis was found in 10 subjects (20.83%), multiple organ failure in 13 subjects (27.08%), severe pancreatitis in 10 subjects (20.83%), AKI in 7 subjects (14.58%), and chronic renal insufficiency in 8 subjects (16.67%). No significant difference was observed between the two groups (P>0.05) (Figure 1).

### Coagulation of filter and venous chamber

In the OG, coagulation grades were distributed as follows: Grade 0 in 19 subjects (39.58%), Grade I in 10 subjects (20.83%), Grade II in 11 subjects (22.92%), and Grade III in 8 subjects (16.67%). In the CG, the distribution was: Grade 0 in 21 subjects (43.75%), Grade I in 12 subjects (25.0%), Grade II in 9 subjects (18.75%), and Grade III in 6 subjects (12.5%). A significant difference was found between the groups (P<0.05).

**Figure 2:**
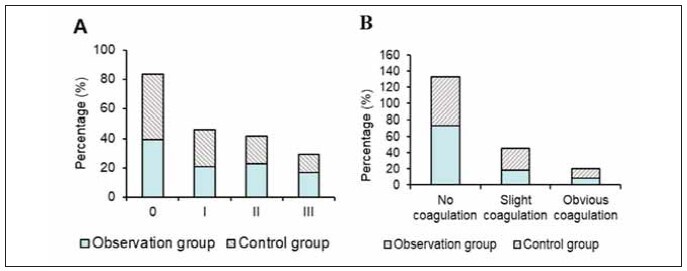
Contrast of coagulation of filter and venous chamber.<br>(Note: a: filter; b: venous chamber coagulation; ^#^ the OG versus the CG, *P*<0.05

Regarding coagulation levels, 35 subjects in the OG (72.92%) showed no coagulation, 9 subjects (18.75%) had mild coagulation, and 4 subjects (8.33%) had significant coagulation. In the CG, 29 subjects (60.42%) showed no coagulation, 13 subjects (27.08%) had mild coagulation, and 6 subjects (12.5%) had significant coagulation. The difference was statistically significant (P<0.05) (Figure 2).

### Coagulation indexes

**Figure 3:**
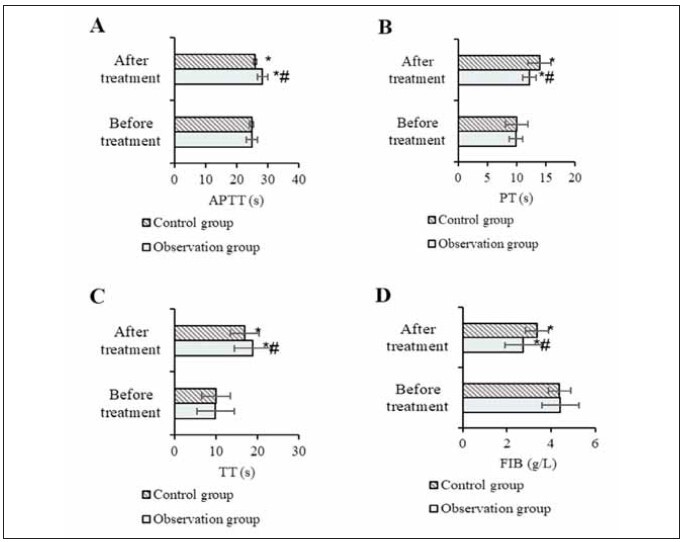
Contrast of coagulation indexes<br>(Note: A: APTT; B: APTT; C: TT; D: FIB; * as against that before treatment, ^#^ the OG versus the CG, *P*<0.05)

Coagulation indexes (APTT, PT, TT, FIB) were significantly altered after treatment. APTT, PT, and TT values increased, while FIB decreased (P<0.05). After treatment, the OG showed a marked difference in coagulation values (APTT, PT, TT) compared to the CG, with a significantly lower FIB value in the OG (P<0.05) (Figure 3).

### Biochemical indexes

The blood pH value in the OG changed from 7.38±0.06 before treatment to 7.46±0.09 after treatment. The CG’s pH changed from 7.32±0.07 to 7.46±0.09 (P<0.05). For HCO_3_^-^, the OG changed from 24.34±3.87 mmol/L before treatment to 26.14±4.08 mmol/L after treatment. In the CG, it changed from 23.45±3.71 mmol/L to 25.42±4.63 mmol/L (P<0.05).

**Figure 4:**
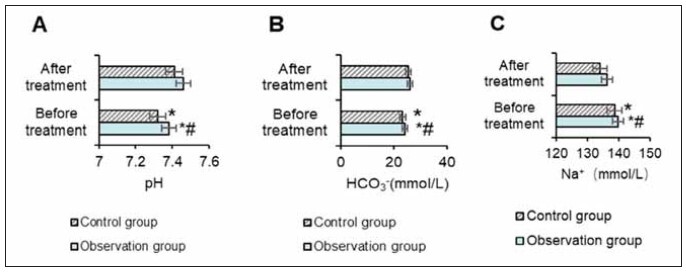
Contrast of biochemical indexes.<br>(Note: A: pH; B: HCO_3_^-^; C: Na^+^; * as against that before treatment, ^#^ the OG versus the CG, *P*<0.05)

Na^+^ levels in the OG decreased from 139.87±5.72 mmol/L before treatment to 136.41±5.63 mmol/L after treatment. In the CG, Na^+^ decreased from 138.72±5.81 mmol/L to 134.13±5.63 mmol/L, with no significant difference between the groups (P>0.05) (Figure 4).

### Blood purification treatment efficiency

In the OG, creatinine (Cr) decreased from 358.58±131.32 mmol/L before treatment to 267.34±94.0 mmol/L, and blood urea nitrogen (BUN) decreased from 21.47±6.75 mmol/L to 13.45±4.08 mmol/L after treatment (P<0.05).

**Figure 5:**
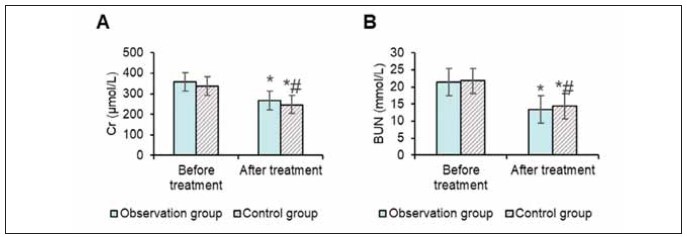
Comparison results of Cr and BUN.<br>(Note: A: Cr; B: BUN; * as against that before treatment, ^#^ the OG versus the CG, P<0.05)

In the CG, Cr decreased from 337.47±142.75 mmol/L to 247.48±124.53 mmol/L, and BUN decreased from 21.73±6.51 mmol/L to 14.38±7.92 mmol/L. The decrease in Cr and BUN in the OG was significantly greater than in the CG (P<0.05) (Figure 5).

### Serum ionised calcium

**Figure 6:**
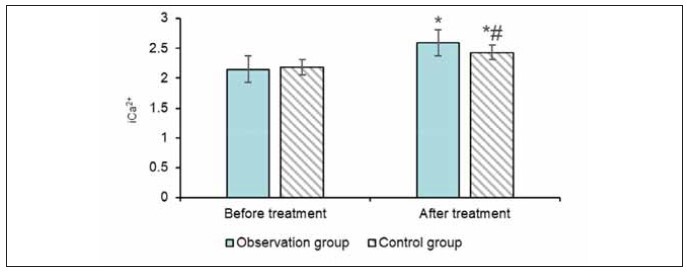
Serum ionised calcium.<br>(Note: ^*^ indicates the CG versus the OG, *P*<0.05)

The OG’s ionised calcium (iCa^2+^) level increased from 2.15±0.13 mmol/L before treatment to 2.59±2.31 mmol/L after treatment. In the CG, iCa^2+^ increased from 2.18±0.15 mmol/L to 2.43±1.89 mmol/L. The increase in iCa^2+^ was significantly higher in the OG than in the CG (P<0.05) (Figure 6).

### Incidence of bleeding

**Figure 7:**
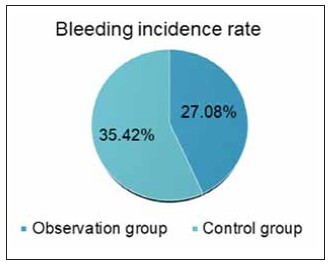
Contrast of bleeding time incidence.<br>(Note: * indicates the CG versus the OG, *P*<0.05)

The incidence of bleeding in the OG was 27.08%, with 13 cases out of 48 subjects, while in the CG, the incidence was 35.42%, with 17 cases out of 48 subjects. The number of bleeding cases was significantly lower in the OG compared to the CG (P < 0.05) (Figure 7).

## Discussion

This research demonstrates that local sodium citrate anticoagulation in continuous renal replacement therapy (CRRT) for children with sepsis significantly reduces the risk of bleeding, maintains blood filter patency, and improves renal function more effectively than heparin-free anticoagulation. These results are consistent with findings from other studies that have highlighted citrate’s advantages over heparin, particularly in critically ill patients with high bleeding risks.

Several studies have shown that citrate anticoagulation can provide safer and more effective management than heparin in CRRT. For instance, previous research emphasised that citrate reduces bleeding risk in critically ill patients and offers better control over coagulation pathways. This study also reported fewer bleeding complications in patients treated with citrate, aligning with our findings that citrate significantly reduces bleeding risks in pediatric sepsis patients undergoing CRRT [15] [16] [17]. The citrate infusion method, which reduces free calcium availability and prevents thrombin activation, is safe and effective, with less impact on overall coagulation than heparin therapy [18] [19].

In terms of coagulation parameters, this study found significant increases in activated partial thromboplastin time (APTT), prothrombin time (PT), and thrombin time (TT) in the overall group (OG) following treatment, accompanied by a notable decrease in fibrinogen (FIB) levels. These changes were more pronounced in the OG compared to the control group (CG), suggesting a stronger anticoagulant effect. Our results are consistent with previous studies, which showed that citrate therapy prolonged APTT and PT more significantly than heparin, with fewer systemic anticoagulation-related complications. These differences can be attributed to citrate’s ability to localise anticoagulation without systemic effects, whereas heparin’s widespread action increases the risk of systemic bleeding and coagulopathy in patients with sepsis [17] [18] [19] [20] [21] [22].

Another key finding of this study is the improvement in renal function, as indicated by the reduction in creatinine (Cr) and blood urea nitrogen (BUN) levels. These results were more prominent in the OG, which used sodium citrate anticoagulation. Previous studies also reported improvements in renal function with citrate anticoagulation, attributed to its less detrimental effect on renal perfusion than heparin. Citrate’s ability to maintain optimal filter patency prevents clot formation and reduces the need for frequent filter replacements, which can mitigate renal damage. Additionally, our results suggest that citrate may protect renal function by minimising the adverse impact of coagulation dysfunction during CRRT [23] [24] [25].

In pediatric patients, particularly those with sepsis, citrate anticoagulation has shown superior benefits in reducing bleeding risks and improving CRRT efficacy. A study demonstrated that sodium citrate was safe and effective in pediatric patients undergoing CRRT for sepsis, reducing the incidence of bleeding complications and maintaining blood filter functionality [26]. Our study supports these findings, showing that local sodium citrate anticoagulation reduces the risk of bleeding and enhances the safety and effectiveness of treatment. The improved renal function and reduced bleeding risks observed in our study may be due to citrate’s more localised action than heparin, which may be particularly advantageous in patients with sepsis who are already at high risk for bleeding.

Another important consideration is the regulation of acid-base balance and electrolyte levels. Our results indicated that the OG experienced more significant changes in pH, bicarbonate (HCO_3_^-^), and sodium (Na^+^) levels compared to the CG, suggesting a more substantial effect of citrate therapy on these parameters. This finding supports the results of previous studies that have highlighted citrate’s beneficial impact on acid-base and electrolyte regulation during CRRT. Citrate anticoagulation may help better maintain the stability of acid-base balance and electrolyte levels during blood purification, which is crucial for patient stability and organ function recovery in septic patients [27].

In contrast to our findings, some studies have suggested that the impact of citrate on acid-base balance can be variable depending on patient characteristics and other complicating factors, such as liver dysfunction. For example, a study observed that in patients with impaired liver function, citrate therapy can lead to metabolic alkalosis due to the accumulation of bicarbonate [23]. However, this issue was not apparent in our study, possibly due to our cohort’s more controlled use of citrate and closer monitoring of acid-base balance.

Regarding alternative anticoagulants, newer agents like apixaban have been explored for CRRT, particularly in pediatric patients. While these newer anticoagulants show promising results in terms of safety and efficacy, their use remains limited due to a lack of extensive clinical data in sepsis patients. In contrast, citrate remains a well-established option for CRRT, with a proven track record of safety and efficacy in critically ill patients, including those with sepsis [28]. Thus, while there is ongoing interest in newer anticoagulants, citrate remains a first-line anticoagulation strategy in CRRT due to its demonstrated benefits in preventing bleeding and improving overall treatment outcomes.

One limitation of this study is its relatively small sample size, which may affect the generalizability of the results to a larger population. Additionally, the study was conducted at a single centre, which could introduce biases related to specific treatment protocols or patient characteristics. The lack of long-term follow-up means that the long-term effects of citrate anticoagulation on patient outcomes, including survival rates and chronic kidney function, were not assessed. Furthermore, the study did not account for potential confounding factors, such as variations in sepsis severity or comorbidities, which could influence the observed outcomes [29].

## Conclusion

In conclusion, this study demonstrates that local sodium citrate anticoagulation in continuous blood purification is a safe and effective alternative to heparin-free anticoagulation, particularly for children with sepsis. Citrate anticoagulation significantly reduces the risk of bleeding, maintains the patency of blood filters, and helps regulate acid-base balance and electrolyte stability. These findings suggest that sodium citrate may offer better therapeutic outcomes, especially in patients with a high bleeding risk. However, further research with larger, multicenter studies and long-term follow-up is needed to understand its efficacy and safety across diverse patient populations fully.

## Dodatak

### Acknowledgements

We would like to thank all the Pediatric Emergency and Intensive Care Unit medical staff at Northwest Women’s and Children’s Hospital and the Department of Pediatrics at Tangdu Hospital, Air Force Medical University, for their assistance in conducting this study. Special thanks to the children and their families for participating in the research.

### Funding

This study received no funding.

### Authors’ contributions

Huixia Wang contributed to the study design, data collection, and analysis. Hongfang Zhao was responsible for the conception of the study, supervision, and manuscript preparation. Both authors reviewed and approved the final manuscript.

### Ethical approval

The Ethics Committee of Northwest Women’s and Children’s Hospital, Xi’an, Shaanxi Province, China, approved the study. Written informed consent was obtained from the parents or guardians of all the children enrolled in the study.

### Data availability statement

The data supporting this study’s findings are available from the corresponding author upon reasonable request.

### Conflict of interest statement

All the authors declare that they have no conflict of interest in this work.
